# rs1234313 and rs45454293 are risk factors of cerebral arterial thrombosis, large artery atherosclerosis, and carotid plaque in the Han Chinese population: a case-control study

**DOI:** 10.1186/s12883-019-1259-9

**Published:** 2019-02-23

**Authors:** Yan Jiang, Xiaomin Liu, Yifeng Du, Shengnian Zhou

**Affiliations:** 1 Department of Neurology, Qilu Hospital of Shandong University and Brain Science Research Institute, Shandong University, 107 Wenhuaxi Road, Jinan, Shandong 250012 People’s Republic of China; 2 Department of Neurology, The First Affiliated Hospital of USTC, Anhui Provincial Hospital, Hefei, 230001 People’s Republic of China; 3 Department of Neurology, Shandong Provincial Hospital, Shandong University, Jinan, 250013 China

**Keywords:** TNFSF4, rs1234313, rs45454293, cerebral arterial thrombosis

## Abstract

**Background:**

Ischemic stroke is a leading cause of mortality and morbidity worldwide. Stenosis or blockage of an artery from atherosclerosis can cause insufficient cerebral blood supply, which leads to ischemic stroke. It has been reported that the polymorphisms of TNFSF4 (tumor necrosis factor super family member 4) are associated with multiple autoimmune diseases. However, it is still unclear whether TNFSF4 gene polymorphisms are associated with ischemic stroke in the Han Chinese population. Here we analyzed the association between TNFSF4 single nucleotide polymorphisms (SNPs) and cerebral arterial thrombosis in the Han Chinese population.

**Method:**

We consecutively recruited 481 patients with cerebral arterial thrombosis and 538 healthy controls. Neck ultrasonography and magnetic resonance imaging (MRI) were used to evaluate large artery atherosclerosis (LAA) and small vessel disease (SVD), as well as the thickness and calcification of carotid artery. DNA was purified from the peripheral blood samples. TNFSF4 SNPs, rs1234313 and rs45454293, were genotyped using PCR.

**Results:**

rs1234313 SNP had a significant correlation with the LAA and SVD subtypes in allelic (G vs A), dominate (GG/GA vs AA) and genotypic (GA vs AA; GG vs AA) models, as well as with the calcification of carotid plaque in dominant (GG/GA vs AA, *p* = 0.022) and genotypic (GA vs AA, *p* = 0.01) models. rs45454293 SNP had a significant correlation with the LAA and SVD subtypes in allelic (G vs A) and genotypic models, as well as with the thick carotid plaque in allelic (G vs A, *p* = 0.01) model.

**Conclusion:**

TNFSF4 SNPs, rs1234313 and rs45454293, are associated with the risk of specific subtypes of cerebral arterial thrombosis in the Han Chinese population.

## Background

Ischemic stroke is a leading cause of mortality and morbidity worldwide [1, 2], especially in low-income and middle-income countries [3]. Ischemic stroke is caused by stenosis or blockage of an artery, which leads to insufficient cerebral blood supply and brain tissue necrosis. Early detection of cerebral arterial thrombosis is critical for ischemic stroke prevention. However, current screening methods for arterial thrombosis are not very efficient, which often result in delayed detection and fail to meet the optimal timing of treatment. Hence, simple and efficient screening methods are highly desirable.

Acute cerebral infarction commonly results from atherosclerosis. Atherosclerosis is a chronic inflammatory disease caused by imbalanced lipid metabolism and a maladaptive immune response. The immune response is mediated by activation and recruitment of immune cells, including T cells and macrophages, to the endothelium of blood vessels. Activated immune cells produce cytokines and inflammatory factors that promote inflammatory reactions [4]. These inflammatory reactions can facilitate the formation of thrombus, and eventually cause ischemia [5, 6]. Studies have shown that activated T cells play an important role in atherosclerosis [6, 7].

TNFSF4 encodes an OX40 ligand (OX40L), a costimulatory molecule involved in T cell activation. TNFSF4 functions as a T cell co-stimulatory molecule [8], and is involved in the formation of atherosclerosis [9, 10]. TNFSF4 is expressed in many other cell types such as macrophages, endothelial cells, and smooth muscle cells [11]. TNFSF4 can activate the inflammatory cells, which in turn secrete cytokines, chemokines, growth factors, matrix metalloproteinase, and clotting factors. Moreover, reports have shown that TNFSF4 polymorphisms are associated with multiple autoimmune diseases, such as Sjogren’s syndrome and systemic lupus erythematosus [12, 13].

Atherosclerosis is caused by the interaction of genetic and environmental factors. Gene polymorphisms play an important role in atherosclerosis [14]. Several SNPs are involved in cardiovascular diseases [15]. SNPs or rare mutations can directly mediate the expression of atherosclerosis-related genes, which in turn participate in the regulation of inflammatory responses. However, few studies have investigated the association of TNFSF4 SNPs and atherosclerosis. Huang et al. demonstrated that TNFSF4 rs1883832 SNP, but not rs1234313 and rs1234314 SNPs, are associated with the risk of ischemic stroke in the Chinese population [16]. Gardener et al. indicated that rs1234313 SNP is associated with carotid plaque phenotypes [17]. Moreover, rs45454293 SNP, a TNFSF4 promoter gene, is considered a risk factor in myocardial infarction in Swedish women [18]. However, it is still unclear whether TNFSF4 gene polymorphisms are associated with the risk of atherosclerosis in the Han Chinese population. In this study we investigated the association of two TNFSF4 SNPs, rs1234313 and rs45454293, with the occurrence of atherosclerosis.

## Method

### Participants

Patients with acute ischemic stroke were recruited at Anhui Provincial Hospital, China, between Oct. 2016 and Apr. 2017. Inclusion criteria: 1) Sudden onset; 2) Local or global dysneuria; 3) No time limits on the duration between the onset and diagnosis; 4) Without cerebral hemorrhage or other lesions [19]. Exclusion criteria: 1) Patients with thrombotic diseases, such as myocardial infarction and homeopathy. 2) Patients who underwent thrombolysis treatment or with intracranial hemorrhage. All healthy controls had a physical examination at Anhui Provincial Hospital. All controls were healthy without angiocardiopathy, and matched the patients in age, gender, and regions. All participants had no consanguinity. The study was approved by the Ethical Committee of Anhui Provincial Hospital, China, and informed consent forms were signed by all participants.

Epidemiological data was collected from all of the participants. The information included, age, gender, region, history of stroke, history of smoking and drinking, and clinical examination parameters. All data was recorded and imported into a computer database.

### Neck ultrasonography

All of the patients with acute cerebral infarction had neck ultrasonography. Atherosclerotic plaque was defined as the ratio of the thickness of blood vessel linings to the peripheral vascular wall greater than 50% in common carotid artery, internal carotid artery, and bifurcation. Maximal carotid plaque thickness (MCPT, mm) was measured from the top of the plaque, and MCPT > 1.9 mm was defined as thick plaque [20]. Signals with strong echoes are used as markers of calcified plaques. The irregularity of sclerosis plaque was analyzed. For each patient, we confirmed the plaque existence and classified the plaque type.

#### TOAST classification

LAA and SVD were analyzed according to the TOAST classification [21, 22]. The following was the diagnostic criteria: 1) LAA: carotid artery occlusion or stenosis was 50% greater than the arterial cross section; the lesion of cerebellum or cerebral cortex, or cortical involvement/subcortical infarction was greater than 1.5 cm. 2) SVD: the maximal diameter of the stroke lesion was smaller than 1.5 cm; atypical lacunar infarction existed in clinic samples, but was not detected by MRI.

### DNA extraction and genotyping assays

Peripheral blood samples from all of the participants was collected with EDTA tubes and stored at − 70 °C. DNA was purified from whole blood using FlexGen Blood DNA Kit (0.1-20 ml) (CW054, CWBIO Co Ltd., Beijing, China) according to the manufacturer’s protocol. TNFSF4 SNPs, rs1234313 and rs45454293 were genotyped using TaqMan SNP Genotyping Assay (Applied Biosystems, Foster City, CA, USA) with KAPA PROBE FAST qPCR Kit Master Mix (2×) (KAPA Biosystems, KK4702). The probes were as follows:

rs45454293-F: TTTAGTGGTAAAGGGTACCTGGTGT,

rs45454293-R: TTTTTCCATGAATGAACAAATGAATAG;

rs45454293-FAM: FAM-AGGCCAGCCACAACCTCAAAGAAA-BHQ,

rs45454293-HEX: HEX-AGGCCAGCCACGACCTCAAAGA-BHQ and.

rs1234313-F: TCAGGACATCCCTGGACCTTC,

rs1234313-R: ACCAGCATCTGTTGCTTCTTGAC;

rs1234313-FAM: FAM-CACTATACATTGCTCAAG-MGB,

rs1234313-HEX: HEX-CACTATACGTTGCTCAA-MGB.

The PCR system (10 μL) included 5 μL PROBE FAST qPCR Kit Master Mix (2×), 0.2 μL primer mix (10 μM), 0.4 μL probe mix (10 μM), 1 μL DNA, 0.2 μL 50x ROX and 4.2 μL ultrapure water. The thermal cycles were pre-incubation at 95 °C for 3 min and the amplification (40 cycles) included 95 °C for 3 s, 60 °C for 20s, and 95 °C for 15 s. The genotyping process was performed by personnel blinded to the experimental groups.

### Statistical analysis

Hardy-Weinberg equilibrium of genotype distribution test was performed using published protocols by Wigginton et al. [23] with Microsoft Excel 2007 software. STATA/SE12.0 (StataCorp LP, TX, USA) was used to analyze the characteristics of patients and controls by student t-test or Chi-Square test. Logistic regression analysis was performed to evaluate the relationship between the genotypes and the risk of acute cerebral infarction using SPSS 19.0 and STATA/SE12.0. The data was expressed as 95% confidence intervals (CI) and odds ratio (OR). *p* < 0.05 was considered statistically significant. Multinomial logistic regression was used to analyze the association of genotypes and clinical features. Age, gender, and smoking status was included as additive covariates.

## Results

We genotyped TNFSF4 SNPs, rs1234313 and rs45454293, in a total of 1016 Han Chinese participants, including 481 patients with acute ischemic stroke and 538 health controls. Because 204 of the 481 patients and 2 of the 538 healthy controls did not have basic information, only 277 patients and 536 healthy controls were included in the analysis of concomitant variables. The participants’ information is listed in Table 1. There were more males in the patient group than in the healthy controls (59.77% vs 43.66%) (*p* < 0.0001). The mean age of the patients was older than the healthy controls (65.8 ± 11.5 vs 58.1 ± 9.8 years, *p* < 0.0001). There was a significantly higher percentage of smokers in the patients (34.65%) compared to the healthy controls (23.65%) (*p* < 0.0001). There was no significant difference in drinking history (*p* = 0.46), suggesting that drinking may not be a risk factor of ischemic stroke. Therefore, we only included age, gender, and smoking status as the covariates in the following analyses. In addition, we also discovered that the patients had a higher rate in morbidity of diabetes (19.4% vs 2.6%, *p* < 0.0001) and hypertension (72.1% vs 38.3%, *p* < 0.0001) than healthy controls. Moreover, most of the clinical parameters, such as systolic pressure and blood glucose, showed a significant difference between the patients and controls. These results demonstrate that the patients with ischemic stroke had abnormal physical signs compared to the healthy controls.

**Table 1:** Characteristics of patients with acute ischemic stroke and healthy controls

Variable	Cases	Controls	*P* value^a^
Numbers	277	536	
Sex			< 0.0001*
Male	153 (256)	234	
Female	103 (256)	302	
Han Ethnicities	277	536	
Smoking status			< 0.0001*
Smokers	70 (202)	128	
Non-smokers	110 (202)	395	
Quit smokers	22 (202)	13	
Drinking status			0.46
Drinker	76 (232)	160 (532)	
Non-drinker	156 (232)	372 (532)	
Diabetes	45 (232)	14 (535)	< 0.0001*
Hypertension	168 (233)	204 (533)	< 0.0001*
			*P* value^b^
Age (year)	65.8 ± 11.5	58.1 ± 9.8	< 0.0001*
Clinic parameters			
Systolic pressure (mmhg)	142.51 ± 18.6 (247)	133.12 ± 11.49	< 0.0001*
Diastolic pressure (mmhg)	85.4 ± 12.15 (247)	79.19 ± 10.88	< 0.0001*
Blood glucose (mmol/L)	6.64 ± 3.45 (238)	5.31 ± 2.14	< 0.0001*
Cholesterol (mmol/L)	4.43 ± 1.24 (238)	4.64 ± 1.18	0.02*
Lipoprotein (low) (mmol/L)	2.41 ± 0.89 (238)	2.55 ± 0.95	0.05
Lipoprotein (high) (mmol/L)	1.09 ± 0.32 (238)	1.17 ± 0.35	0.007*
Triglyceride (mmol/L)	1.66 ± 0.9 (238)	1.68 ± 1.02	0.74

^a^Two-sided χ^2^ test; ^b^Two-Sample T test; ^*^P < 0.05 indicating a significant difference

The summary of genotype distribution and allele frequency of rs1234313 and rs45454293 SNPs is shown in Table 2. The genotype distribution of rs1234313 and rs45454293 SNPs in both the patients and controls were consistent with the Hardy-Weinberg equilibrium (rs1234313: *p* = 0.96 and 0.28; rs45454293: *p* = 0.07 and 0.29, respectively).

**Table 2:** Genotype and Allele Frequencies of the TNFSF4 rs1234313 and rs4545293 polymorphism in patients with acute ischemic stroke and healthy controls

rs1234313	Cases (%), *n* = 481	Controls (%), *n* = 538
Genotypes		
AA	212 (44.07%)	230 (42.75%)
GA	215 (44.5%)	252 (46.84%)
GG	54 (11.23%)	56 (10.41%)
Alleles		
A	639 (66.42%)	712 (66.17%)
G	323 (33.58%)	364 (33.83%)
rs4545293	Cases (%), *n* = 481	Controls (%), *n* = 538
Genotypes		
AA	3 (0.62%)	6 (1.12%)
GA	112 (23.28%)	125 (23.23%)
GG	366 (76.09%)	407 (75.65%)
Alleles		
A	118 (12.27%)	137 (12.73%)
G	844 (87.73%)	939 (87.27%)

*TNFSF4* tumor necrosis factor super family member 4

### rs1234313 SNP analysis

rs1234313 SNP showed a distribution of 44.07%/44.5%/11.23% for AA/GA/GG genotypes in the patients, and 42.75%/46.84%/10.41% in the controls, respectively. There was no significant difference in the frequency of allele A and G between the patients and controls (Allele A: 66.42% vs 66.17%; allele G: 33.58% vs 33.83%, χ^2^ = 0.02, *p* = 0.9).

We further analyzed the relationship of rs1234313 SNP with the susceptibility of ischemic stroke (Table 3). When the covariates (age, gender, and smoking status) were excluded, there was no significant association of genotypic or allelic rs1234313 SNP with ischemic stroke (*p* = 0.599 and *p* = 0.904, respectively). There was no significant difference in the dominant or recessive model (*p* = 0.670 and *p* = 0.675, respectively). Next, we performed the same analysis with the inclusion of the covariates (age, gender, and smoking status). There was no significant difference in the four genetic models (Table 3).

**Table 3:** The association of TNFSF4 rs1234313 and rs4545293 polymorphisms with acute ischemic stroke

	Comparison	Covariates	OR	*P*-Value
Model (rs1234313)				
Allele	G vs A	none	0.99	0.94
Genotypic	GA vs GG vs AA	none	NA	0.67
Dominant	GG/GA vs AA	none	0.94	0.68
Recessive	GG vs GA/AA	none	1.16	0.53
Allele	G vs A	Sex, ages and smoking status	1.00	0.95
Genotypic	GA vs GG vs AA	Sex, ages and smoking status	NA	0.82
Dominant	GG/GA vs AA	Sex, ages and smoking status	0.99	0.98
Recessive	GG vs GA/AA	Sex, ages and smoking status	1.11	0.67
Model (rs4545293)				
Allele	G vs A	none	1.03	0.86
Genotypic	GA vs GG vs AA	none	NA	0.86
Dominant	GG/GA vs AA	none	1.56	0.59
Recessive	GG vs GA/AA	none	1.03	0.85
Allele	G vs A	Sex, ages and smoking status	1.02	0.91
Genotypic	GA vs GG vs AA	Sex, ages and smoking status	NA	0.57
Dominant	GG/GA vs AA	Sex, ages and smoking status	1.07	0.94
Recessive	GG vs GA/AA	Sex, ages and smoking status	1.04	0.86

*TNFSF4* tumor necrosis factor super family member 4

Next, we evaluated the association of rs1234313 SNP with different types of carotid plaques in patients with ischemic stroke (Table 4). Recessive genetic model was not evaluated because all of the recessive genotypes were less than other genotypes. We found that rs1234313 SNP had a significant correlation with carotid plaque calcification using dominant (RR = 0.73, *p* = 0.022) and genotypic (GA vs AA, RR = 0.638, *p* = 0.01) models, indicating that AA genotype of rs1234313 SNP was associated with a high risk of carotid plaque calcification in patients with ischemic stroke. No significant association was discovered between the rs1234313 SNP and the types of plaques (single plaque, multi plaque, thick plaque, and irregular plaque).

**Table 4:** Association of TNFSF4 rs1234313 polymorphism with types of carotid plaque in patients with ischemic stroke

Genetic model	Comparison	RRR^a^ vs. controls	*p*-value	95% CI lower	95%CI Upper
Single plaque					
allelic	G vs A	0.995	0.971	0.750	1.320
Dominant	GG/GA vs AA	0.873	0.346	0.659	1.157
genotypic	GA vs AA	0.756	0.149	0.517	1.105
	GG vs AA	1.313	0.381	0.713	2.417
Multi plaque					
allelic	G vs A	0.994	0.954	0.813	1.216
Dominant	GG/GA vs AA	0.943	0.534	0.783	1.135
genotypic	GA vs AA	0.898	0.352	0.715	1.127
	GG vs AA	1.143	0.602	0.691	1.893
Thick plaque					
allelic	G vs A	1.176	0.216	0.909	1.522
Dominant	GG/GA vs AA	1.141	0.234	0.918	1.417
genotypic	GA vs AA	1.139	0.335	0.875	1.482
	GG vs AA	1.490	0.241	0.765	2.900
Calcification					
allelic	G vs A	0.804	0.112	0.615	1.052
Dominant	GG/GA vs AA	0.730	0.022*	0.557	0.956
genotypic	GA vs AA	0.638	0.010*	0.452	0.900
	GG vs AA	0.912	0.767	0.469	1.677

*TNFSF4* Tumor necrosis factor super family member 4, *RRR* relative risk ration, *CI* Confidence interval; *, *p* < 0.05, indicating a significant difference

**Fig. 1:**
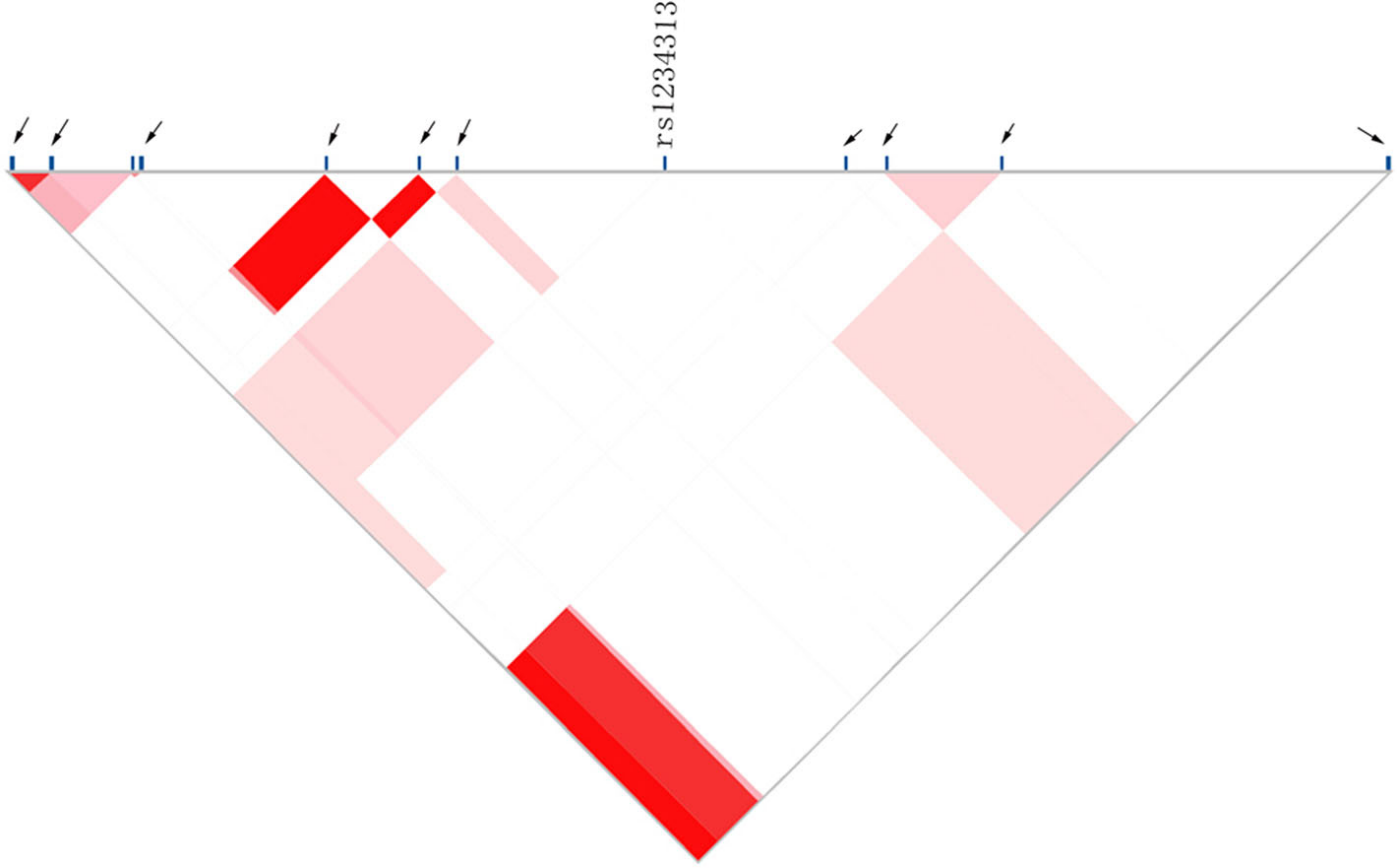
The linkage disequilibrium analysis of rs1234313 SNPs. Arrows indicate the SNPs from 2000 bp up-stream to 2000 bp down-stream of rs1234313. The pattern shows an association between two SNPs, colors range from light to dark, indicating the LD value r^2^ was from low to high. The red color represents *r*^*2*^ = 1 (complete LD). SNPs, single nucleotide polymorphisms. TNFSF4, tumor necrosis factor super family member 4. LD, linkage disequilibrium

Linkage analysis of rs1234313 SNP was carried out in the Han Chinese population. The outcomes are shown in Fig. 1. We found that there were 12 SNPs from the up-stream 2000 bp to down-stream 2000 bp of rs1234313. However, none of these SNPs had a strong linkage disequilibrium association with rs1234313, indicating that linkage equilibrium was within the range. These results further demonstrate the independent inheritance of rs1234313.

### rs45454293 SNP analysis

The distributions of rs45454293 SNPs AA/GA/GG genotypes were 0.62%/23.28%/76.09% in patients and 1.12%/23.23%/75.65% in the controls. There was no significant difference in the frequency of allele A and G between the patients and controls (A:12.27% vs 12.73%; G: 87.73% vs 87.27%, χ^2^ = 0.101, *p* = 0.751).

We further analyzed the association of rs45454293 SNP with the susceptibility of ischemic stroke (Table 3). We found that there was no significant association between rs45454293 SNP and ischemic stroke with or without the covariates (age, gender and smoking status) (Table 3).

We also analyzed the association of rs45454293 SNP with the types of carotid plaques in patients with ischemic stroke (Table 5). We found that allele A of rs45454293 SNP was associated with a decreased risk in stroke patients with thick carotid plaque using allelic model (RR = 1.076, *p* = 0.01). No other significance was found in other types of carotid plaques.

**Table 5:** Association of TNFSF4 rs4545293 polymorphism with types of carotid plaque in patients with ischemic stroke

Genetic model	Comparison	RRR^a^ vs. controls	*p*-value	95% CI lower	95%CI Upper
Single plaque					
allelic	G vs A	1.047	0.158	0.982	1.115
Dominant	GG/GA vs AA	1.003	0.852	0.975	1.031
genotypic	GA vs AA	0.999	0.992	0.862	1.158
	GG vs AA	1.004	0.800	0.971	1.039
Multi plaque					
allelic	G vs A	0.994	0.827	0.940	1.051
Dominant	GG/GA vs AA	0.993	0.621	0.968	1.020
genotypic	GA vs AA	0.973	0.626	0.872	1.086
	GG vs AA	0.991	0.622	0.958	1.026
Thick plaque					
allelic	G vs A	1.076	0.010*	1.018	1.137
Dominant	GG/GA vs AA	1.002	0.890	0.973	1.032
genotypic	GA vs AA	0.977	0.825	0.792	1.204
	GG vs AA	1.004	0.800	0.971	1.039
Calcification					
allelic	G vs A	0.990	0.760	0.926	1.058
Dominant	GG/GA vs AA	0.998	0.893	0.971	1.026
genotypic	GA vs AA	0.996	0.936	0.894	1.108
	GG vs AA	0.997	0.881	0.962	1.034

*TNFSF4* Tumor necrosis factor super family member 4, *RRR* relative risk ration, *CI* Confidence interval; *, *p* < 0.05, indicating a significant difference

**Fig. 2:**
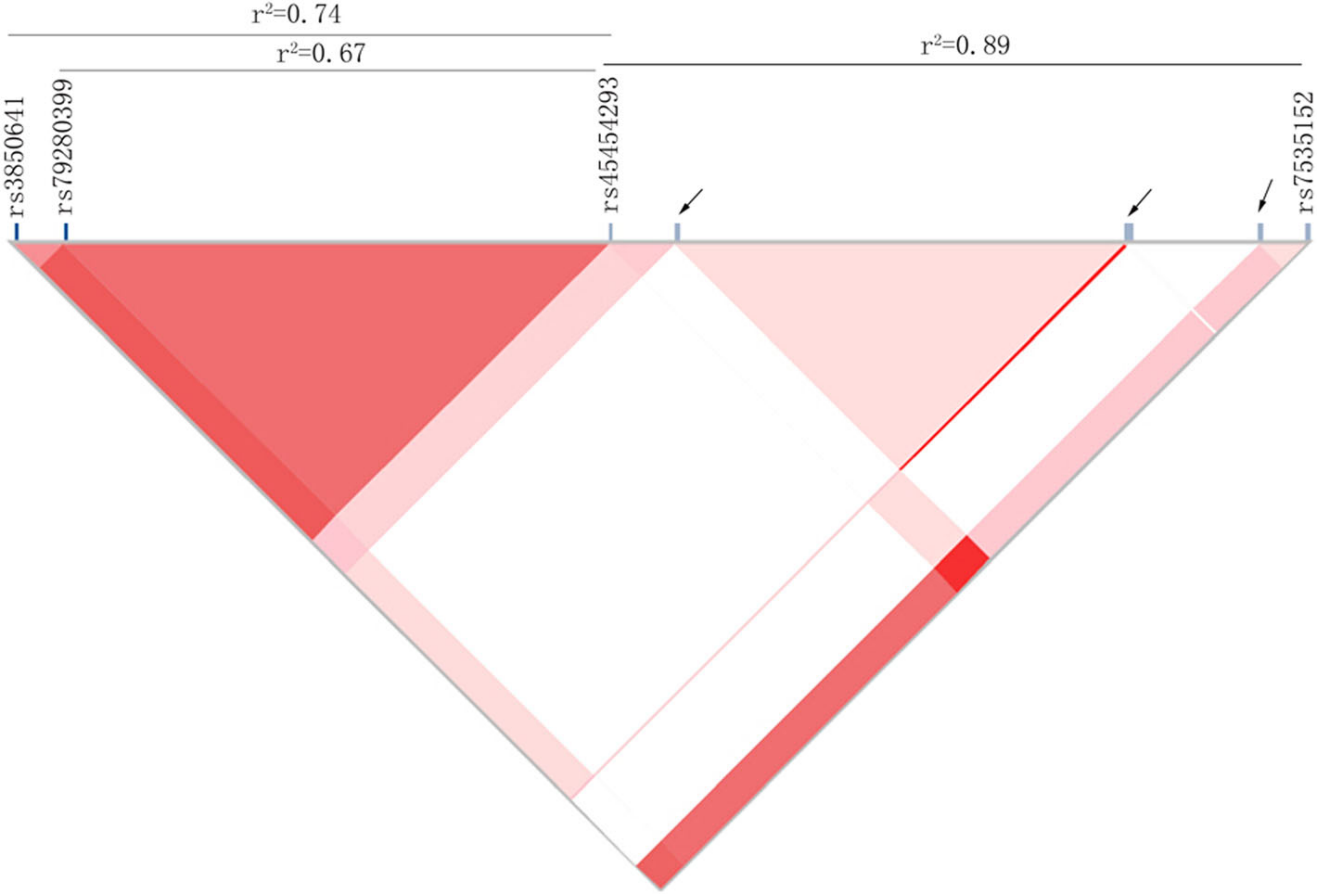
The linkage disequilibrium analysis of rs45454293 SNPs. Arrows indicate the SNPs from 2000 bp up-stream to 2000 bp down-stream of rs45454293. r^2^ was used to detect the LD level (*r*^*2*^ = 1 indicates a complete LD) between two SNPs. SNPs, single nucleotide polymorphisms. TNFSF4, tumor necrosis factor super family member 4. LD, linkage disequilibrium

Linkage analysis of rs45454293 SNP is shown in Fig. 2. We found that there were 8 SNPs from the up-stream 2000 bp to down-stream 2000 bp of rs45454293. Moreover, 3 (rs7535152, rs3850641, rs79280399) of them had a LD relationship with rs45454293 (*r*^*2*^ ≥ 0.5), indicating that rs45454293 SNP is accompanied with other SNPs.

#### LAA and SVD subtype analysis

There were 138 patients with LAA and 97 patients with SVD. We found that rs1234313 was greatly associated with the risk of both LAA and SVD in all genetic models (Table 6), indicating that allele G, GG and GA might be protective compared to allele A and AA genotypes. We also found that rs45454293 was greatly associated with both LAA and SVD only in the allelic model (G vs A) (Table 6). When compare to allele A, the allele G was a risk factor in LAA and a protective factor in SVD.

**Table 6:** The association of TNFSF4 rs1234313 and rs4545293 polymorphism with LAA and SVD in patients with ischemic stroke

Genetic model	Comparison	RRR^a^ vs. controls	*p*-value	95% CI lower	95%CI Upper
rs1234313					
LAA					
allelic	G vs A	0.397	0.000*	0.337	0.467
Dominant	GG/GA vs AA	0.580	0.000*	0.502	0.670
genotypic	GA vs AA	0.543	0.000*	0.455	0.650
	GG vs AA	0.228	0.000*	0.150	0.346
SVD					
allelic	G vs A	0.388	0.000*	0.319	0.473
Dominant	GG/GA vs AA	0.543	0.000*	0.451	0.654
genotypic	GA vs AA	0.486	0.000*	0.383	0.616
	GG vs AA	0.241	0.000*	0.153	0.382
rs45454293					
LAA					
allelic	G vs A	1.087	0.000*	1.051	1.125
Dominant	GG/GA vs AA	1.011	0.131	0.997	1.025
genotypic	GA vs AA	1.026	0.643	0.920	1.144
	GG vs AA	1.014	0.089	0.998	1.031
SVD					
allelic	G vs A	0.917	0.020*	0.853	0.987
Dominant	GG/GA vs AA	0.993	0.633	0.963	1.023
genotypic	GA vs AA	1.002	0.970	0.916	1.095
	GG vs AA	0.985	0.526	0.941	1.032

*TNFSF4* Tumor necrosis factor super family member 4, *LAA* large artery atherosclerosis, *SVD* small vessel disease, *RRR* relative risk ration, *CI* Confidence interval; *, *p* < 0.05, indicating a significant difference

## Discussion

In this study we demonstrate that there are significant differences in age, gender, smoking status, and most of the clinic parameters, such as diabetes, and hypertension between patients with ischemic stroke and the healthy controls. The differences in age, gender, and smoking status between the patients and the controls imply that these factors might be risk factors for ischemic stroke. Therefore, we included these factors as variables in logistic analyses. We analyzed the risk factors of ischemic stroke in all genetic models with or without concomitant variables (age, gender, and smoking status). Therefore, our analyses reflect the association between TNFSF4 polymorphism and stroke.

We discovered that all the genetic models including allelic, dominant and genotypic models of rs1234313 were greatly associated with LAA and SVD, suggesting that allele G, GG or GA might reduce the risk of LAA and SVD, compared to A and AA genotypes. In addition, rs45454293 had a reverse effect on the risk of LAA and SVD in the allelic models (G vs A). These results indicate different mechanisms between LAA and SVD. Meanwhile, ischemic stroke might be regulated by distinct SNPs of the TNFSF4 gene. No study has reported the relationship between TNFSF4 SNPs and LAA or SVD subtypes of ischemic stroke. Our study provides a better understanding of the mechanisms of stroke.

We also discovered that the dominant (GG/GA vs AA) and genotypic (GA vs AA) models of rs1234313 SNPs had a significant correlation with carotid plaque calcification in patients with acute ischemic stroke. Moreover, the allelic model (G vs A) of rs45454293 SNP has a significant correlation with the thick carotid plaque in patients with acute ischemic stroke. These results are consistent with the study by Gardener et al. [17], which indicates that rs1234313 is associated with the carotid plaque phenotypes in stroke patients. We also investigated the association between carotid plaque calcification and atherosclerotic cerebral infarction, which is not included in the study by Huang et al. [16].

Carotid plaque can be used as an important indicator for the diagnosis of atherosclerosis, cardiovascular and cerebrovascular diseases. Hence, the type of carotid plaques is a critical diagnostic criterion for patients with cerebral arterial thrombosis. We demonstrated that rs1234313 and rs45454293 SNPs have a significant correlation with carotid plaque calcification and thick carotid plaque, respectively, in patients with cerebral arterial thrombosis. Therefore, TNFSF4 SNPs can be used as an indicator to define the population with increased susceptibility to ischemic stroke, and to achieve early detection/prevention in order to decrease the morbidity of ischemic stroke.

Studies have shown that TNFSF4 rs2205960 and rs844648 SNPs are associated with the susceptibility to systemic sclerosis [24]. Meanwhile, a meta-analysis study demonstrates that TNFSF4 rs2205960 SNP may confer susceptibility to SLE (systemic lupus erythematosus) in different populations and that the TNFSF4 rs1234315 SNP is associated with the susceptibility to SLE in Asian [25] and Malaysian populations [26]. Lian et al., [27] demonstrated that rs844648 and rs704840 SNPs of TNFFS4 are associated with an increased risk of NMOSD (Neuromyelitis optica spectrum disorders) in different genetic models, indicating that cerebrovascular diseases are mediated by multiple factors. To date, there is no report on TNFSF4 SNPs in Chinese patients with ischemia stroke. Our study is the first that demonstrates these two TNFSF4 SNPs might be associated with carotid plaque calcification and thick plaque. In addition, we have excluded all patients who had previously received any type of treatment. Therefore, our findings can be used for predicting the risk of disease onset in patients without treatments.

In addition, TNFSF4 expression is associated with an increased risk of atherosclerosis. Two independent human cohorts studies demonstrated that TNFSF4 is expressed in antigen-presenting cells at human carotid atherosclerotic lesions [28]. Interestingly, potent immune mediators, CD40 and CD40L, are overexpressed in experimental and human atherosclerotic lesions. The interruption of CD40/CD40L not only can diminish the formation and progression of atheroma in mice, but it can also foster changes in lesion biology and structure, which are associated with plaque stabilization in patients [9]. The receptor/ligand interactions play a central role in atherosclerosis, hence TNFSF4 / TNFRSF4 interaction/interrupt can be used as a new treatment method for atherosclerosis [9].

In this study we demonstrate that two TNFSF4 SNPs (rs1234313 and rs45454293) have a significant correlation with the acute ischemic stroke. However, there are some limitations of this study. Firstly, nearly half of the patients did not have the basic information, which resulted in the exclusion of these patients from the concomitant variable analyses. If we had more samples, the allelic and dominant genetic models of rs1234313 might show a statistical significance. Secondly, we did not analyze the relationship between these two SNPs, although both of them were detected in stroke patients. Lastly, the association between ischemic stroke and other features of carotid plaque was not analyzed. In our future study we will include other features, such as ulcerative surface, echolucent core, and thin fibrous cap.

## Conclusion

We investigated the relationship between TNFSF4 SNPs, rs1234313 and rs45454293, with acute ischemic stroke in 481 patients and 538 health controls. We did not find any significant differences between patients and controls based on the four genotypic model analyses (Dominate (GG/GA vs AA); genotypic (GA vs AA); Recessive (GG vs GA/AA); Allele (G vs A)) with or without concomitant variables (age, gender and smoking status). However, we found that rs1234313 has a significant correlation with LAA and SVD in all genotypic models; rs45454293 has a significant correlation with LAA and SVD in allelic and genotypic models. In addition, we discovered that rs1234313 SNPs has a significant correlation with carotid plaque calcification using dominant and genotypic models; while rs45454293 SNPs have a significant correlation with thick carotid plaque using allelic model analysis. Linkage disequilibrium analysis shows that rs45454293 SNPs has a strong LD association with other SNPs. These results indicate that these two TNFSF4 SNPs can be used as indicators in the screening of populations susceptible to ischemic stroke.

## Abbreviations

CI: Confidence intervals
CT: Computed tomography
LD: Linkage disequilibrium
MCPT: Maximal carotid plaque thickness
MRI: Magnetic resonance imaging
NMOSD: Neuromyelitis optica spectrum disorders
OR: Odds ratio
OX40L: OX40 ligand
SNPs: Single nucleotide polymorphisms
TNFSF4: Tumor necrosis factor super family member 4
